# Development and initial psychometric evaluation of the computer-based prostate Cancer screening decision aid acceptance scale for African-American men

**DOI:** 10.1186/s12874-019-0776-y

**Published:** 2019-07-10

**Authors:** Otis L. Owens, Nikki R. Wooten, Abbas S. Tavakoli

**Affiliations:** 1 University of South Carolina, College of Social Work, 1514 Pendleton Street, Columbia, SC 29208 USA; 2 University of South Carolina, College of Nursing, 1601 Greene Street, Columbia, SC 29208 USA

**Keywords:** Statistical factor analysis, Technology acceptance, Computer-assisted decision making, Prostatic neoplasms, Culturally appropriate technology, African Americans

## Abstract

**Background:**

To reliably evaluate the acceptance and use of computer-based prostate cancer decision aids (CBDAs) for African-American men, culturally relevant measures are needed. This study describes the development and initial psychometric evaluation of the 24-item Computer-Based Prostate Cancer Screening Decision Aid Acceptance Scale among 357 African-American men.

**Methods:**

Exploratory factor analysis (EFA) with maximum likelihood estimation and polychoric correlations followed by Promax and Varimax rotations.

**Results:**

EFA yielded three factors: Technology Use Expectancy and Intention (16 items), Technology Use Anxiety (5 items), and Technology Use Self-Efficacy (3 items) with good to excellent internal consistency reliability at .95, .90, and .85, respectively. The standardized root mean square residual (0.035) indicated the factor structure explained most of the correlations.

**Conclusions:**

Findings suggest the three-factor, 24-item *Computer-Based Prostate Cancer Screening Decision Aid Acceptance Scale* has utility in determining the acceptance and use of CBDAs among African-American men at risk for prostate cancer. Future research is needed to confirm this factor structure among socio-demographically diverse African-Americans.

## Background

Prostate cancer (PrCA) incidence and mortality rates are higher among African-American men than any other racial group [1]. Many socio-economic [2, 3], environmental [3, 4], and epigenetic [5–7] factors are hypothesized to be key contributors to PrCA disparities among African-Americans and other racial groups, but no definitive causal links have been identified between PrCA and these factors. Fraught with controversy [8], the public health response to PrCA has potentially contributed to PrCA disparities. Whereas clear screening recommendations are available for many other major cancers (e.g., breast, colon, lung, cervical), recommendations for PrCA are not clear cut and have evolved over the past two decades [9, 10]. Until recently, the U. S. Preventive Services Task Force [11] recommended against routine prostate specific antigen (PSA) screening, a position that was counter to other organizations such as the American Cancer Society [12] and American Urological Association [13], which recommend that men make an informed decision with a healthcare provider about whether to receive PrCA screening. In 2017, the U. S. Preventive Services Task Force released draft recommendations that are more consistent with agencies that support informed decision making [14], which involves a man understanding the risks, benefits, uncertainties, and alternatives to PrCA screening and participating in the decision at the level that he desires [15].

In order to engage in informed decision making, African-American men need plain language PrCA knowledge information and adequate decision self-efficacy [16]. PrCA knowledge refers to the information necessary for an individual to understand PrCA, including the prostate’s anatomy and function; PrCA risk factors; types of PrCA screening and the risk benefits, uncertainties, and alternatives to each type of screening; and PrCA warning signs [17]. Self-efficacy refers to the level of confidence an individual possesses to actively involve himself, to the extent that he desires, in the PrCA screening decision-making process [18]. Prior studies indicate that African-American men who participate in informed decision-making interventions for PrCA screening often experience increased PrCA knowledge and decision self-efficacy [19], which may equip them to actively participate in informed decision making. Increasingly, PrCA decision-making interventions are being offered through digital mediums such as computers and mobile phones [20–22]. Technology-based dissemination strategies may, in part, be driven by stark increases in technology ownership [23] across all racial, ethnic, and age groups. However, older African-Americans with low incomes and low education attainment are the least likely to have access to computer or mobile technologies [23, 24].

To determine the quality of a user’s experience while using a technology-based PrCA intervention, some researchers conduct feasibility or usability testing [20–22]. However, most studies evaluating the efficacy of technology-based interventions assessed the target population’s technology acceptance, which encompasses the conditions under which an individual will adopt a technology for regular use [25]. Therefore, technology acceptance is one key determinant of the sustainability of a technology-based intervention beyond its research use. Sustainability is especially important for PrCA screening interventions because informed decisions about screening will occur many times over a man’s life course and the effects of the intervention after one exposure to the intervention could diminish over time [26].

### Technology acceptance and use models

While some technology acceptance factors are highly correlated with usability (e. g., ease of use; [27]), most technology acceptance models also include constructs that are external to the user (e. g., social influences such as subjective norms; [25]). Based on socio-ecological theory, technology acceptance models posit that an individual’s decision to adopt a specific technology is not based solely on self-identified benefits, but is the result of complex interactions between an individual and their social and physical environment [25]. One of the most widely accepted models informing the measurement of technology acceptance is the Technology Acceptance Model [28], which posits that users’ adoption of a technology for normal use is dependent on its perceived usefulness (i.e., enhances task performance) and perceived ease of use (i.e., extent to which a technology requires effort). Developed in 1989, the technology acceptance model has undergone several modifications to enhance its utility (e.g., Technology Acceptance Model 2; [29, 30]). Specifically, modified models have integrated a plethora of other socio-ecologic factors that influence technology use. [30] Since it’s introduction, the technology acceptance model has been tested with over 25 various external variables that are posited to influence the relationship between the three major constructs: perceived usefulness, perceived ease of use, and technology acceptance [30]. The most commonly tested variables include computer anxiety, self-efficacy, enjoyment, computer support, and computer-use experience [30]. To enhance the performance of the technology acceptance model, Venkatesh and Davis, the model’s creators, have also expanded the model integrating additional five variables, including job relevance (an individual’s perception regarding the degree to which the target system is applicable to his or her job), subjective norms (an individual’s perception that most people who are important to them think they should or should not perform the behavior in question), image (the degree to which use of an innovation is perceived to enhance one’s status in one’s social group), output quality (how well the system performs specific tasks), and result demonstrability (tangibility of the results of using the innovation) to explain the conditions under which individuals affect a users’ perceived ease of use [29]. Two additional variables, experience and voluntariness of use, were hypothesized to mediate subjective norms and perceived usefulness and/or an individual’s intention to use a system. Similar to the original technology acceptance model, this modified version (i.e., Technology Acceptance Model 2) has been widely adopted [25] and used in a variety of contexts, which also includes the assessment of technology-use in the healthcare environment [31, 32]. Synthesizing the Technology Acceptance Model with existing models to examine technology acceptance (e. g., Diffusion of Innovation; [33]), Venkatesh and Davis et al., [34], collaborators on the Technology Acceptance Model 2, developed the Unified Theory of Acceptance and Use of Technology. Developed in 2003, the model postulates that four factors (performance expectancy, effort expectancy, social influence, and facilitating conditions) and four moderators (i.e., age, gender, experience, and voluntariness) determine an individual’s intention to use a technology and ultimately whether they decide to adopt a technology for regular use [34]. In their development of the Unified Theory of Acceptance and Use of Technology, Venkatesh et al. [29] also examined an individual’s self-efficacy, anxiety, and attitudes and hypothesized that these factors were not causally related to technology use intention, but were fully mediated by other factors in their model (e.g., effort expectancy). Based on the Unified Theory of Acceptance and Use of Technology, Venkentash et al. [29] created a 24-item scale, the *Unified Theory of Acceptance and Use of Technology Scale*, that measures acceptance and use of a technology based on each of the aforementioned moderating factors and intention to use technology. In their seminal article, Venkentash et al. [29] found that factor loadings for each item were acceptable with most loadings being .70 or higher. In addition, internal consistency reliability for the full scale and subscales ranged between .77 and .94. As hypothesized by Vekentash et al. [29], self-efficacy, anxiety, and attitudes towards technology did not have a direct causal relationship with intention to use technology. However, exploratory factor analysis of the *Unified Theory of Acceptance and Use of Technology Scale* found that self-efficacy and anxiety are significant predictors of technology use intention [35]. Despite these differences, the *Unified Theory of Acceptance and Use of Technology Scale* has been validated for use across several sectors including, but not limited to, mobile banking, social networking, web-based learning environments, decisions support systems, digital learning environments, and retail [36, 37].

Related to decision support systems and web-based learning environments, there are a growing number of studies that have employed the *Unified Theory of Acceptance and Use of Technology Scale* for assessing technology acceptance in health environments [38]. These studies most often investigate the acceptance of clinical support technology, such as electronic medical records, by healthcare providers or other clinical staff [39–43]. However, a number of studies assessed the acceptance of web-based telecare systems among patients [44–47]. Each of these studies demonstrate the application of the Unified Theory of Acceptance and Use of Technology to healthcare settings, though the strength of hypothesized relationships among the variables has varied. According to Holden and Karsh [31], these inconsistencies in the strength of associations among variables can be largely explained by differences in the contextual operationalization of the constructs within the Unified Theory of Technology Use and Acceptance. More specifically, some studies implement the scale with general wording, but others often alter wording to be context specific to the type of technology being tested [31]. The addition of contextual relevancy is necessary to ensure that the scale, which was originally designed for use in non-healthcare settings will meaningfully translate for use in a healthcare setting [31]. Therefore, some studies also added environment-related constructs to the model which made it even more contextually relevant [31, 44, 46–49]. For example, Ciperman et al. [46] posited that a doctor’s opinion regarding the use of telehealth technology influences performance expectancy, computer anxiety influences effort expectancy, and perceived security influences performance and effort expectancy and has a direct influence on behavioral intention. Findings show that computer anxiety has a significant negative influence on effort expectancy, while doctor’s opinion and performance expectancy had significant positive influences on performance expectancy and behavioral intention, respectively. Despite methodological differences, the aforementioned studies report acceptable to high internal consistency reliability scores (α= > .70), with some researchers conducting factor analyses [46, 50]. A few studies also found that the Unified Theory of Acceptance and Use of Technology Scale had strong convergent and divergent validity for assessing technology acceptance for health-related purposes [46, 50, 51].

Only one recent study [52] has investigated the Unified Theory of Technology Use and Acceptance in relation to cancer care or decision making. Among 300 cancer survivors, Senft et al. [52] examined whether facilitating conditions, social influence, ease of use, perceived usefulness, and security/trustworthiness was associated with eHealth use and if attitudes about the security and trustworthiness of online health services was associated with eHealth use more strongly among African-American than White cancer survivors. They discovered that facilitating conditions and perceived usefulness are associated with increased eHealth use among African-Americans and Whites and social influence did not influence e-health use among either group. Also, perceived ease of use was associated with decreased e-health activity for Whites only, whereas security/trustworthiness is associated with increased eHealth activity for African-Americans only. The authors do not report the internal consistency reliability of the *Unified Theory of Technology Use and Acceptance Scale* in this study. A second cancer-related study [53], proposes to use the Unified Theory of Technology Use and Acceptance Scale to understand the acceptance and use of an mHealth app for PrCA survivorship by patients, caregivers, and clinicians in the United Kingdom, but only a study protocol is currently available.

Although a number of studies report acceptable reliability and validity of the *Unified Theory of Technology Use and Acceptance Scale* for assessing technology use and acceptance across sectors, including healthcare, the psychometric properties of this scale have not been tested among African Americans. Having a culturally-relevant measure of technology use and acceptance is needed to measure the performance of technology-based interventions that seek to enhance decision making about PrCA screening among African-American men, who experience the highest mortality from the disease [1]. Given the need for and utility of a reliable and valid CBDA for PrCA, this study described the development and tested the psychometric properties of the *Computer-Based Prostate Cancer Screening Decision Aid Acceptance Scale* among African-American men.

## Methods

This psychometric study used cross-sectional data from a pilot study conducted to assess the efficacy of *iDecide*, a computer-based decision aid (CBDA) to prepare African-American men for informed decision making about PrCA screening. During the pilot study, participants received a self-administered, paper survey at post-intervention to measure their acceptance of *iDecide*. Detailed recruitment strategies and a description of *iDecide* are published in prior manuscripts [54]. Human subject’s approval was received from the Institutional Review Board at the University of South Carolina.

### Participants

This study included a purposive sample of 354 African-American men aged 40 and older. Eligible participants were those who (a) self-identified as African American; (b) spoke and comprehended English; (c) had no personal history of PrCA; and (d) had no self-reported history of cognitive decline. Participants were not required to have prior technology use experience. They were recruited from several social and faith-based venues in South Carolina between July 2015 to February 2016. All participants received a written consent form in-person prior to their participation in any study activities. Prior to requesting a signature on the consent form, the form was explained in detail by a member of the research team. This study was approved by the Institutional Review Board at the University of South Carolina.

### Scale development

The *Unified Theory of Acceptance and Use of Technology Scale* was adapted to examine the usability and acceptance of *iDecide*, a CBDA for African-American men [55]. Specifically, each question was modified to refer generally to a CBDA as opposed to generally referring to a *“*system”. For example, question Q28 (Table 1) was adapted to read “*The system is somewhat intimidating to me*” to “*The CBDA is somewhat intimidating to me*.” This question revision is similar to prior studies that have adapted the *Unified Theory of Acceptance and Use of Technology Scale* to be contextually relevant to their study environment [31]. Scale modification was conducted by the first author (O.O), an African-American male with expertise in health communications, health technology, and PrCA within the target population. Therefore, scale items were adapted for contextual relevancy while maintaining content equivalence and items were eliminated that were not contextually related to the CBDA (e.g., *The senior management of this business has been helpful in the use of the system*). Subscales (performance expectancy, effort expectancy, social influence, and facilitating conditions, self-efficacy, attitudes towards technology, and anxiety) and two of four original moderators (i.e., age, experience) that are hypothesized to be directly related to technology acceptance were retained. Because this scale is specific to CBDAs, the modified scale was titled, the *Computer-Based Prostate Cancer Screening Decision Aid Acceptance Scale*.

**Table 1:** Computer-based prostate cancer screening decision aid acceptance scale

Domain	Text of original questions	Text Of Revised Questions (Question Number)
Performance Expectancy	1. I would find the system useful in my job	1. I find the CBDA useful (Q1)
	2. Using the system enables me to accomplish tasks more quickly	2. Using the CBDA enables me to find information on prostate cancer more quickly (Q2)
	3. Using the system increases my productivity	3. Eliminated Question
	4. If I use the system, I will increase my chances of getting a raise	4. Using the CBDA increases my chances of learning about prostate cancer (Q3)
		5. Using the CBDA increases my likelihood of finding information on prostate cancer (Q4)
Effort expectancy	5. My interaction with the system would be clear and understandable	6. My interaction with the CBDA is clear and understandable (Q5)
	6. It would easy for me to become skillful at using the system	7. It is easy for me to become an expert at using the CBDA (Q6)
	7. I would find the system easy to use	8. I find the CBDA easy to use (Q7)
	8. Learning to operate the system is easy for me.	9. Learning to use the CBDA was easy for me (Q8)
	9. Using the system is a good idea	10. Using the CBDA is a good idea (Q9)
Attitudes Toward Technology	10. The system makes study more interesting	11. The CBDA makes learning about prostate cancer more interesting (Q10)
	11. Studying with the system is fun.	12. Learning about prostate cancer with the CBDA is fun (Q11)
	12. I like studying with the system	13. I like using the CBDA (Q12)
Social influence	13. People who influence my behavior think that I should use the system	14. Eliminated Question
	14. People who are important to me think that I should use the system	15. People who are important to me will likely support my use of the CBD (Q13)
	15. The senior management of this business has been helpful in the use of the system	16. Eliminated Question
	16. In general, the organization has supported the use of the system	17. Eliminated Question
Facilitating Conditions	17. I have the resources to use the system	18. Eliminated Question
	18. I have the knowledge necessary to use the system	19. I have the knowledge necessary to use the CBDA (Q14)
	19. The system is not compatible with other systems that I use	20. The CBDA is not compatible with other systems I use (Q15)
	20. A specific person (or group) is available for assistance with system difficulties	21. Eliminated Question
Self-Efficacy	21. I can complete a job or task using the system if there was no one around to tell me what to do	22. I can complete the prostate cancer education program using the CBDA if there is no one around to tell me what to do as I go (Q16)
	22. I can complete a job or task using the system if I can call someone for help if I get stuck	23. I can complete the prostate cancer education program using the CBDA if I can call someone for help if I get stuck (Q17)
	23. I can complete a job or task using the system if I have a lot of time to complete the job	24. I can complete the prostate education program using the CBDA if I have a lot of time to complete the program (Q18)
	24. I can complete a job or task using the system if I have just the built-in help facility for assistance.	25. I can complete the prostate education program using the CBDA if I have just reviewed instructions provided by the avatar (Q19)
	25. I feel apprehensive about using the system	26. I feel nervous about using the CBDA (Q20)
Anxiety	26. It scares me to think that I could lose a lot of information using the system by hitting the wrong key	27. It scares me to think that I could lose my place in the prostate cancer program by hitting the wrong key on the CBDA (Q21)
	27. I hesitate to use the system for fear of making mistakes I cannot correct	28. I hesitate to use the CBDA for fear of making mistake I cannot correct (Q22)
	28. The system is somewhat intimidating to me	29. The CBDA is somewhat intimidating to me (Q23)
Behavioral intention to use the system	29. I intend to use the system in the next x months	30. I intend to use the CBDA in the future (Q24)
	30. I predict that I will use the system in the next x months.	31. Eliminated question
	31. I plan to use system in the next x months.	32. Eliminated question

The *Computer-Based Prostate Cancer Screening Decision Aid Acceptance Scale* is comprised of 24-items with Likert scale response categories ranging from 1 (*strongly agree*) to 5 (*strongly disagree*) and 6 (*does not apply*). Scoring involves taking the average of all responses, excluding *does not apply* responses. A higher score indicates greater acceptance and use of the CBDA for PrCA informed decision making. Table 1 compares items of the *Unified Theory of Acceptance and Use of Technology Scale* to items of the *Computer-Based Prostate Cancer Screening Decision Aid Acceptance Scale*.

### Conceptual framework of the computer-based prostate Cancer screening decision aid acceptance scale

Based on the Unified Theory of Technology Use and Acceptance [29],the *Computer-Based Prostate Cancer Screening Decision Aid Acceptance Scale* emphasizes the influence of the dynamic interplay between the individual and their social and physical environment about whether an individual will adopt a CBDA. To determine the acceptance and use of a CBDA for PrCA informed decision making, seven factors are influential including: (a) performance expectancy (the degree to which an individual believes that the CBDA will lead to personal gains such as increases in PrCA knowledge and decision self-efficacy), (b) effort expectancy (the amount of effort associated with using the CBDA to retrieve PrCA information), (c) social influence (the degree to which an individual perceives that his or her social network will endorse the use of the CBDA), (d) facilitating conditions (the degree to which an individual believes that infrastructure exists to support their use of a CBDA for informed decision making about PrCA screening), (e) computer anxiety (the level of emotional fear or apprehension when an individual thinks about having to use a CBDA for finding PrCA information), (f) self-efficacy (an individual’s belief in their ability to effectively use the CBDA to find PrCA information, (g) attitudes towards technology (an individual’s positive or negative behaviors regarding use of the CBDA) and (h) behavioral intention (an individual’s intention to use a technology). Each of these factors are moderated by an individual’s age and technology-use experience [34]. Counter to the original Unified Theory of Technology Use and Acceptance, gender and voluntariness are not moderators in our conceptual framework because the CBDA is designed for an African-American male population and use of this technology is voluntary.

### Pre-testing

To assess face validity, the *Computer-Based Prostate Cancer Screening Decision Aid Acceptance Scale* was pre-tested with a convenience sample of two African-American men with high school or higher education. During the pre-test, the men were provided with a survey containing the full battery of 65-items that were used during the testing of the *iDecide* PrCA screening CBDA. These men completed a hard copy survey and noted if there were questions, words, or concepts on the survey they found difficult to interpret or that might be difficult to interpret for men with low reading levels. After survey completion, they were interviewed by the first author (O.O.) about difficulties completing the survey along with questions about survey formatting (e.g., clarity of instructions, response option formatting). Neither participant suggested changes to the survey.

### Data analysis

Descriptive statistics described the sociodemographic characteristics of African-American men in the sample. Polychoric correlations assessed the association between factors and subscale items. Cronbach’s alpha assessed internal consistency reliability for the total scale and each subscale.

Exploratory factor analysis (EFA) is a data-driven, exploratory technique and that does not require a priori specification of the relationships between latent and observed variables [56–58]. Thus, model specification is not required because factor structure and factor loadings are assumed to be unknown. In this study, EFA was conducted to identify the number of latent constructs (factors) and the underlying factor structure of the *Computer-Based Prostate Cancer Screening Decision Aid Acceptance Scale*. Performance expectancy, effort expectancy, social influence, facilitating conditions, computer anxiety, self-efficacy, and attitudes towards technology were exogenous latent variables hypothesized to be correlated with each other. Subscale items loading on each factor were also hypothesized to be correlated with each other. The number of participants to item ratio was 14:1, which is above the recommended 10:1 often used to determine a priori sample size for EFA [59].

EFA was conducted using preliminary estimates of communalities obtained from the square of the multiple correlation coefficient of each variable. Iterated principal factor extraction with prior communalities set to 1 was used for data extraction followed by Varimax rotation. Factor retention was assessed through parallel analysis, which has been demonstrated as a more accurate assessment of factor retention than other factor retention methods [58]. Specifically, using the K-1 method can lead to sampling error, which can overestimate the number of factors [60]. Parallel analysis produces correlation matrices from a randomly chosen simulated dataset that has a similar number of observations as the original dataset [60]. The simulated observations have the same potential sampling error as the original observations. Eigenvalues were then computed for both the simulated and original data. To determine the number of factors to retain, simulated and original data eigenvalues were compared to determine the point at which the eigenvalue in the simulated data was higher than the original data [61]. The number of factors before this transition point denoted the number of factors that were retained [61]. A scree plot was also produced to visually compare eigenvalues from simulated and original data to corroborate our determination of the number of factors to retain [59].

Factor loadings were assessed using item communalities, cross-loadings, and item statistics. A factor with less than three factor loadings was considered weak and unstable [59] and was deleted from the factor structure. Factors with three or more factor loadings were retained. An item was determined to load on a factor if the factor loading was 0.40 or greater for that factor and was less than 0.40 for other factors [62]. An item was cross-loaded if it loaded on more than one factor at 0.40 or above [59]. The standardized root mean square residual measured the difference between the observed correlation and the predicted correlation. Acceptable standardized root mean square residual estimates are less than .05 [63]. The Kaiser-Meyer-Olkin measured sampling adequacy and estimates between 0.8 and 1 were considered adequate [64].

Missing values ranged from 0.56% (*n* = 2) to 2.54% (*n* = 9) for scale items. Single and multiple imputation were used to impute missing values for 24 items. Means for each item was compared with and without imputation, which included no imputation, single imputation, and multiple imputation (*n* = 1000) for missing data, which were similar. Descriptive statistics were analyzed using original data (*N* = 354). EFA was conducted using original, single, and multiple imputation datasets given the *Computer-Based Prostate Cancer Decision Aid and Acceptance Scale* is a major adaptation of the *Unified Theory of Acceptance and Use of Technology Scale*. All data analyses were performed using SAS/STAT® statistical software, version 9.4 [65].

## Results

For each item of the 24-item scale, mean responses ranged from 2.2 to 4.46. Table 2 reports sociodemographic characteristics of the 354 African-American men. They had a mean age of 59.5 ± 9.61 years and most were married (55%, *n* = 194). An overwhelming majority were insured (91%; *n* = 323) and had a regular healthcare provider (87%, *n* = 309). Nearly half were employed (47%, *n* = 167) or reported a household income between $20,000–79,999 (46%, *n* = 165). Over half (54%, *n* = 192) had a high school diploma or attended some college. Most participants report using technology prior to study participation including: television (87%, *n* = 307), cellphones (85%, *n* = 303), automated teller machines (71%, *n* = 252), computers (69%, *n* = 246) and/or tablet computers (57%, *n* = 201). They also reported using the following technology features: cell phone apps (75%, *n* = 76), text messaging (69%, *n* = 243), email (67%, *n* = 237), and/or the internet (65%, *n* = 229).

**Table 2:** Summary of African American male participant characteristics

Characteristics			*N* = 354
Age in years, mean ± SD			59.5 ± 9.61
Marital status, n (%)			
Single/Never married			73 (20.62)
Married			194 (54.81)
Unreported			87 (24.57)
Employment Status			
Employed			167 (47.10)
Retired			94 (26.55)
Unemployed			84 (23.73)
Unreported			9 (2)
Annual household income 2014			
Less than $20,000			100 (28.24)
$20,000 - $79,999			165 (46.61)
≥ $80,000			75 (21.15)
Unreported			14 (4)
Number of people supported by income			
1			103 (30.29)
2			117 (31.41)
3+			87 (28.60)
Unreported			33 (9.70)
Education			
Less than high school			37 (10.45)
High school/GED or some college			192 (54.23)
Bachelor’s degree or higher			117 (33.06)
Unreported			8 (2.26)
Health Insurance status			
Insured			323 (91.24)
No coverage			31 (8.76)
Regular Health			
Regular Healthcare Provider			
Yes			309 (87.28)
No			45 (12.72)
Which of the following technologies have you used?	Television	307	(86.72)
	ATM	252	(71.18)
	Cell Phone	303	(85.59)
	Computer	246	(69.49)
	Touch-Screen Tablet	201	(56.77)
Which of the following technology features have you used?	Cell Phone App	266	(75.14)
	Texting	243	(68.64)
	Email	237	(66.94)
	Internet	229	(64.68)

### Factor structure of the computer-based prostate Cancer decision aid acceptance scale

**Fig. 1:**
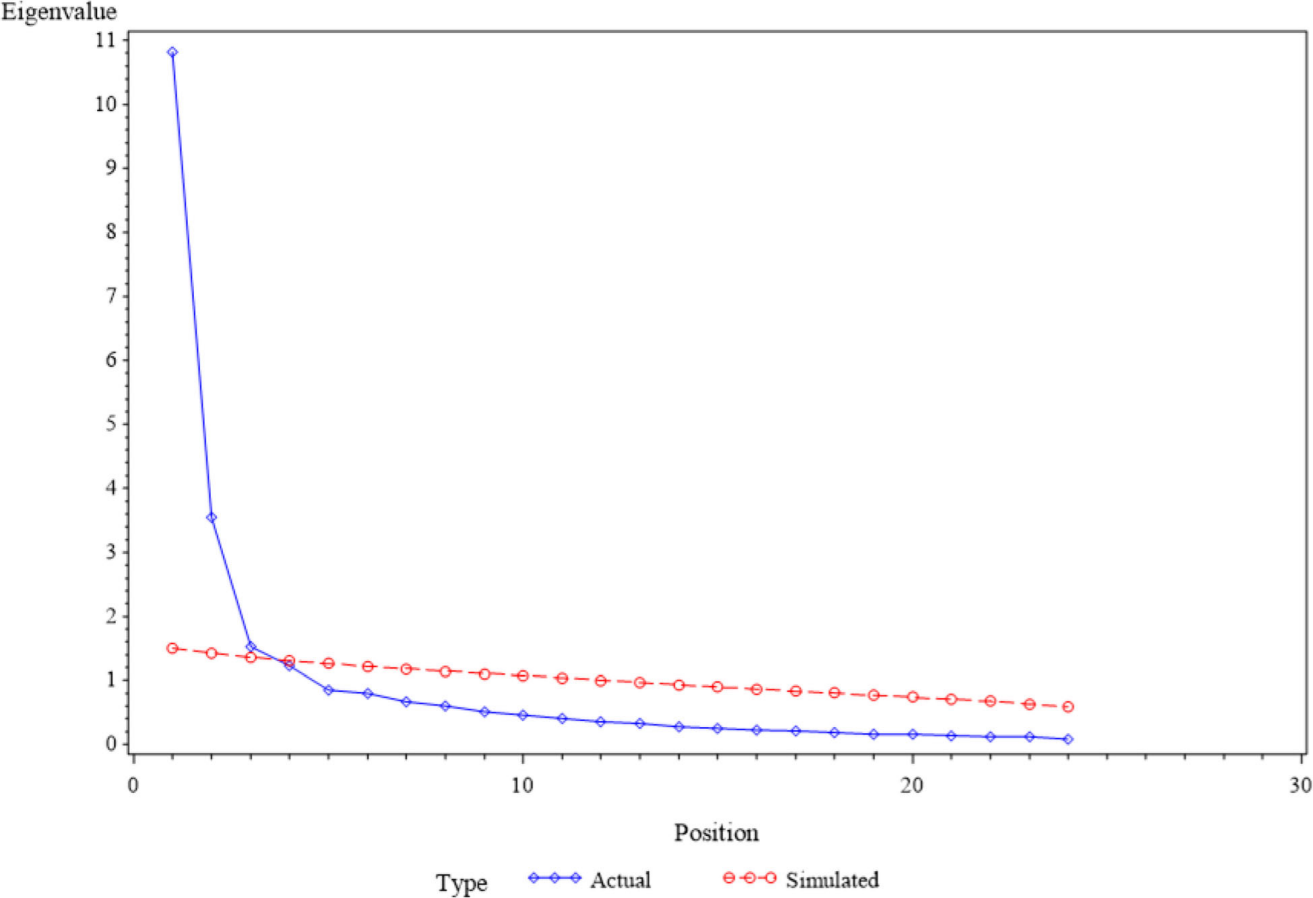
Scree plot for the Computer-Based Prostate Cancer Screening Decision Aid Acceptance Scale. Actual: refers to the eigenvalues in the original dataset. Simulated: refers to the eigenvalues in the randomly chosen simulated dataset

Table 3 reports factor loadings of the 24-item *Computer-Based Prostate Cancer Screening Decision Aid Acceptance Scale* using the original, single and multiple imputation datasets. Factor loadings were similar for original, single and multiple imputation datasets. Parallel analysis, scree plot (Fig. 1), and the proportion of variance explained by each factor suggested three meaningful factors. Factor 1 (F1), *Technology Use Expectancy and Intention* had 16 factor loadings ranging from .51 to .85 for original data, and .44 to .83 and .44 to .83 for single and multiple imputation, respectively. Factor 2 (F2), *Technology Use Anxiety*, had five factor loadings ranging from .52 and .92 for original data, and .41 to .93 for both single and multiple imputation. Factor 3 (F3), *Technology Use Self-efficacy*, had three factor loadings ranging from .67 to .88 for original and imputed datasets.

**Table 3:** Factor structure and factor loadings of the 24-item computer-based prostate cancer screening decision aid acceptance scale, with and without imputation (*N* = 354)

Items	Without Imputation (Original)			Single Imputation			Multiple Imputation (1000)		
	Factor 1	Factor 2	Factor 3	Factor 1	Factor 2	Factor 3	Factor 1	Factor 2	Factor 3
Q4	85			82			82		
Q7	83			83			83		
Q3	83			82			82		
Q1	82			78			78		
Q2	82			78			82		
Q5	82			82			82		
Q8	81			79			79		
Q12	74		41	70		41	70		41
Q14	72			69			69		
Q6	70			68			68		
Q13	63		43	61		43	61		43
Q9	63			61		42	61		42
Q10	61			61		44	61		44
Q11	58		46	56		47	56		47
Q16	54			52			52		
Q24	51			44			44		
Q22		92			93			93	
Q21		92			91			91	
Q23		91			91			91	
Q20		89			87			87	
Q15		52			41			41	
Q18			88			78			78
Q17			81			74			74
Q19			67			57			57

*Note*: Dataset without imputation is the original data. Factor 1 = Technology Use Expectancy and Intention. Factor 2 = Technology Use Anxiety. Factor 3 = Technology Use Self-Efficacy. Factor loadings are equivalent to standardized regression coefficients between scale items and factors. Internal consistency reliability for the total scale is 0.91 for no imputation, and 0.89 for both single and multiple imputation. Internal consistency reliability is 0.95, 0.90, and 0.85 for Factors 1, 2, and 3, respectively for no, single, and multiple imputation. Root Mean Square Residual = 0.035 and Kaiser-Meyer-Olkin Measure of Sampling Adequacy = 0.93 for without imputation (original), single and multiple imputation

Factor-loadings varied between original and imputed datasets. For the original dataset, three items, Q11, Q12, and Q13, cross-loaded on two factors—*Technology Use Expectancy and Intention* (F1) and *Technology Use Self-Efficacy* (F3). For single and multiple imputed datasets, five items, Q9, Q10, Q11, Q12, and Q13, cross-loaded on two factors—*Technology Use Expectancy and Intention* (F1) and *Technology Use Self-Efficacy* (F3). All cross-loaded items were retained on *Technology Use Expectancy and Intention* (F1) because all factor loadings were highest on F1 compared to F3 (Table 3).

The Kaiser-Meyer-Olkin measure of sampling adequacy was 0.93, which is acceptable. All residuals were small and the overall standardized root mean square residual was 0.035, indicating that the factor structure explains most of the correlations. Further, the internal consistency reliability of the full scale was .91 for the original dataset and .89 for both single and multiple imputation datasets; and .95, .90, and .85 for Factors 1, 2, and 3, respectively, for all datasets (Table 4).

**Table 4:** Internal consistency reliability of the computer-based prostate cancer screening decision aid scale (*N* = 354)

Factors	Without Imputation (Original)	Single Imputation	Multiple Imputation
Technology Use Expectancy and Intention	0.95	0.95	0.95
Technology Use Anxiety	0.90	0.90	0.90
Technology Use Self-Efficacy	0.85	0.85	0.85
Total Scale	0.91	0.89	0.89

*Note.* Factor 1 = Technology Use and Expectancy and Intention. Factor 2 = Technology Use Anxiety. Factor 3 = Technology Use Self-Efficacy

## Discussion

This study evaluated the psychometric properties of the 24-item, *Computer-Based Prostate Cancer Screening Decision Aid Acceptance Scale*, in African-American men using a CBDA for informed PrCA screening decision making. EFA resulted in a 24-item, three-factor structure involving *Technology Use Expectancy and Intention* (F1), *Technology Use Anxiety* (F2), and *Technology Use Self-Efficacy* (F3). Factor loadings were moderate to high and the total scale and subscales had good to excellent internal consistency reliability. These findings expand the Unified Theory of Technology Acceptance and Use and builds upon prior evidence indicating that technology acceptance and use is a multidimensional construct. To our knowledge, this is the first scale developed to measure the efficacy of a CBDA for PrCA screening. Our findings suggest the *Computer-Based Prostate Cancer Screening Decision Aid Acceptance Scale* has utility in determining the acceptance and use of CBDAs by African-American men for informed PrCA screening decision making.

Although our findings are theoretically consistent with the Unified Theory of Technology Acceptance and Use, the three-factor structure of the *Computer-Based Prostate Cancer Screening Decision Aid Acceptance Scale* is inconsistent with the six-factor structure the *Unified Theory of Acceptance and Use of Technology Scale* [29]. Our findings are more parsimonious and suggest that some of the Unified Theory of Acceptance and Use of Technology constructs may be highly correlated when used for informed PrCA screening decision making by African-American men. Specifically, Venkatesh et al.’s [29] validation study did not find that self-efficacy, computer anxiety, and attitudes towards technology were correlates of technology acceptance and use. However, the factor structure of the *Computer-Based Prostate Cancer Screening Decision Aid Acceptance Scale* showed that performance expectancy (Q1-Q4), effort expectancy (Q5-Q8), attitudes toward technology (Q9-Q12), social influence (Q13) and behavioral intention to use the system (Q24) loaded on *Technology Use Expectancy and Intention* with factor loadings ranging from .44 and .85 for all datasets. The high factor loadings and factor loading pattern suggest that these constructs are far more interrelated than distinctive in a community sample of middle-aged African-American men who used a measure that was adapted for healthcare decision making compared to individuals from business, non-profit, and academic organizations who used the *Unified Theory of Acceptance and Use of Technology Scale* to assess use and acceptance of a new technology in prior psychometric studies. Our data are also distinct from two healthcare-related studies that conducted psychometric testing [46, 50] of the *Unified Theory of Acceptance and Use of Technology Scale* for assessing healthcare technology acceptance among non-African-Americans. For example, in the assessment of factors influencing Korean healthcare professionals’ adoption of mobile electronic medical records, Kim et al. [50] validated the six-factor structure posited by Venkentash et al. [29], but this population is innately different from our target population of African-American end-users. Additionally, our purposive sample of African-American men, recruited from social and faith-based organizations in a southeastern state, may be more homogeneous in terms of age, socioeconomic status, and belief systems, which may partially support Sundaravej’s (2010) suggestion that socio-demographic factors (e.g., age, experience) moderate an individual’s intention to use a specific technology. These moderators, such as faith which has been shown to positively influence technology acceptance [66, 67], were not tested in our study. Furthermore, social influence and behavioral intention were measured with one item, which may have forced them to load onto factors that are not directly related. Future research should confirm the factor structure of the *Computer-Based Prostate Cancer Screening Decision Aid Acceptance Scale*, test moderators, and assess model fit using more fit indices. Overall, the Kaiser-Meyer-Olkin and standardized root mean square residual estimates and small residuals further support the good fit of our 24-item, three-factor model.

As for factor loadings, one of the two items for facilitating conditions (Q14; “*I have the knowledge necessary to use the CBDA.”*) loaded on *Technology Use Expectancy and Intention* (F1), whereas the other item (Q15; ‘*The CBDA is not compatible with other systems I use.*) loaded on *Technology Use Anxiety* (F2). Specifically, Q14 is related to whether an individual has the “knowledge” necessary to use the CBDA, whereas Q15 is related to “compatibility” of the CBDA with technologies that an individual currently uses. This suggests that facilitating conditions may not be a distinct construct and possibly conceptually related to technology use and intention overall, especially in the context of informed PrCA screening decision making. Among middle-aged African-American men who are using CBDAs to assist with PrCA screening decisions, facilitating conditions may be different compared to assessment for diffusion of innovation in non-profit, business, and academic organizations. The lack of homogeneity of facilitating conditions for technology use and intention may also explain why the items loaded on two different factors [68]. Further, given African Americans have low levels of technology experience overall [69], African-American men in this study may lack the technical knowledge to ascertain whether the CBDA was consistent with technology they currently use. Future administrations and psychometric testing of the *Computer-Based Prostate Cancer Screening Decision Aid Acceptance Scale* in a more heterogeneous sample of African-American men who are at risk for PrCA and women who assist with PrCA screening decision making may provide more evidence about the role of facilitating conditions for technology use and acceptance, especially regarding age, gender, and social environment.

The four computer anxiety (Q20-Q23) items loaded onto *Technology Use Anxiety* (F2). Counter to the *Unified Theory of Technology Use and Acceptance Scale*, computer anxiety was a salient correlate that may predict whether an African-American man decided to use a specific technology. Prior studies have also identified anxiety as detrimental to intention to use technology For example, among 1204 racially diverse participants, Czaja et al. [69] found that lower computer anxiety, higher education, younger age, higher computer use self-efficacy, and higher intelligence were associated with higher technology use, and that African Americans have less experience overall with technology, which may indicate lower computer self-efficacy and higher levels of computer anxiety. Similarly, among 300 older adults (64 to 98 years), Mitzner et al. [70] found that the strongest correlates of positive perceptions about technology use were computer attitudes (i.e., self-efficacy, anxiety, and interest), more technology experience, and agreeable personalities. Because computer anxiety is a prominent influencer of technology use, our findings expand the conceptualization of the Unified Theory of Technology Use and Acceptance to African Americans who may have high levels of computer anxiety and is consistent with current evidence on technology use.

Interestingly, 3 of 4 self-efficacy items (Q17-Q19) loaded onto *Technology Use Self-efficacy* (F3) with factor loadings ranging from .57 to .88, whereas the remaining item (Q16; ‘*I can complete the prostate cancer education program using the CBDA if no one is around to tell me what to do as I go’*) loaded on *Technology Use Expectancy and Intention* (F1) with factor loadings at .54 and .52 for original and imputed datasets, respectively*.* Because African Americans may have less technology use experience and lower technology self-efficacy [69], the idea of using the CBDA without assistance may be anxiety-provoking and increase the effort exerted to use CBDAs for African-American men faced with PrCA screening decision making. Although the Q16 factor loading is contrary to prior evidence on the *Unified Theory of Technology Use and Acceptance Scale* [29], it is consistent with current evidence on technology use among African Americans [69, 70]. The loading of Q16 onto *Technology Use Expectancy and Intention* may also be attributed to question wording. Similar to a reverse worded item, Q16 contains a negation ‘no one around,’ which is different from other self-efficacy items. Therefore, Q16 may have been misinterpreted because of the negation or vague wording, which increases response bias. Perhaps rewording Q16 to read, ‘*I can complete the prostate cancer education program using the CBDA without assistance*’, in future administrations and psychometric testing will reduce response bias and provide further evidence of convergent validity (i.e., items loading on a single factor at 0.50 and above [58]).

Five items (Q9-Q13) cross-loaded on *Technology Use Expectancy and Intention* (F1) and *Technology Use Self-Efficacy* (F3) for both original (Q11-Q13) and imputed (Q9-Q13) datasets. Although all cross-loaded items loaded highest on (see Table 3) and were allocated to *Technology Use Expectancy and Intention* (F1), cross-loading could be the result of vague and/or confusing question wording. Q9, which reads, ‘*Using the CBDA is a good idea*’ and Q10, which reads, ‘*The CBDA makes learning about prostate cancer more interesting’*, may both be related to performance expectancy, social influence, and self-efficacy. Q11, which reads, ‘*The CBDA makes learning about prostate cancer fun,’*; Q12, which reads, ‘*I like using the CBDA*,’; and Q13, which reads, *‘People who are important to me will likely support my use of the CBDA’* may all seem vague and conceptually unrelated to technology use and acceptance for healthcare decision making. Given cancer is a grave topic and that the purposive sample of African-American men were at risk for PrCA, asking about whether learning about PrCA is fun may seem awkward or even inappropriate. Perhaps rewording Q9-Q13 during future administrations of the *Computer-Based Prostate Cancer Screening Decision Aid Acceptance Scale* may improve factor structure.

Study strengths included a large community sample of African American men, which exceeded the minimum recommended sample size for EFA (> 200). However, the African- American men were from one mid-sized city in a southeastern state and may be more homogenous than a national sample of African-American men. Therefore, psychometric findings reported may not be generalizable to African-American men who reside in other U. S. regions or men of other races and ethnicities. Although participants had moderate experience with technology use, they had the least prior experience with using a tablet computer (57%, *n* = 201), the device on which our CBDA was administered. Two items (Q15, Q24) had factor loadings of less than .50 in at least one dataset, which suggests poor convergent validity. Cross-loadings suggest factors may not be conceptually distinct. The modified scale was only pre-tested with two African-American men who may have not been representative of African-American men included in the sample. Lastly, the research team did not test the influence of important moderators that could affect technology acceptance such as faith. Despite these limitations, this study provides valuable psychometric evidence, which can contribute to the future development and evaluation of culturally-tailored CBDAs to facilitate PrCA screening decisions of African-American men who are at risk for the deadliest PrCA globally.

Future psychometric testing (i.e., CFA) is warranted to confirm convergent and discriminate validity of the *Computer-Based Prostate Cancer Screening Decision Aid Acceptance Scale.* Future research should also confirm the factor structure of the *Computer-Based Prostate Cancer Screening Decision Aid Acceptance Scale* using a larger and more demographically diverse sample of African Americans. Having a diverse sample is especially important given that the Unified Theory of Acceptance and Use of Technology postulates that sociodemographic factors such as age and computer experience can moderate technology use and acceptance outcomes.

## Conclusion

In sum, the three-factor, 24-item *Computer-Based Prostate Cancer Screening Decision Aid Acceptance Scale* can be considered distinct from the *Unified Technology Acceptance and Use Scale* given that the later was developed to assess technology acceptance and use in business and banking, whereas the former is a major adaption to assess technology acceptance and use for informed PrCA decision making that may have life or death consequences. Thus, the emotion and anxiety evoked by informed PrCA screening decision making as well as the personal nature of the task including involving family members and health care providers suggest our scale is conceptually unique for healthcare decision making. Although preliminary, psychometric evidence from this study suggests the *Computer-Based Prostate Cancer Screening Decision Aid Acceptance Scale* has a conceptually distinct factor structure, good to excellent internal consistency reliability, and acceptable convergent and discriminant validity. PrCA is highly prevalent among African American men and PrCA knowledge is critical to making decisions about PrCA screening and early identification of PrCA. Given high rates of PrCA mortality among African-American men and the growing development of culturally-tailored CBDAs to assist African-American men with healthcare decisions about PrCA, the *Computer-Based Prostate Cancer Screening Decision Aid Acceptance Scale* can be influential in the evaluation of technology-based PrCA interventions. Most notably, our scale has robust psychometric proprieties for use among African-American men, who are not well-represented in current studies on technology use and acceptance. Because technology is more accessible than ever before and has been integrated into all aspects of our lives, the *Computer-Based Prostate Cancer Screening Decision Aid Acceptance Scale* shows promise as playing a key role in increasing PrCA knowledge and assisting in informed PrCA screening decision making among African-American men.

## Data Availability

The datasets used and/or analysed during the current study are available from the corresponding author on reasonable request.

## Abbreviations

CBDA: Computer-based decision aid
EFA: Exploratory factor analysis
PrCA: Prostate cancer
